# The assessment of carotid intima media thickness and serum Paraoxonase-1 activity in Helicobacter *pylori *positive subjects

**DOI:** 10.1186/1476-511X-9-92

**Published:** 2010-08-30

**Authors:** Halide S Akbas, Sebahat Basyigit, Inci Suleymanlar, Didem Kemaloglu, Serkan Koc, Fatih Davran, Ibrahim Demir, Gultekin Suleymanlar

**Affiliations:** 1 Akdeniz University Faculty of Medicine, Department of Biochemistry, Antalya, Turkey; 2 Akdeniz University Faculty of Medicine, Department of Medicine, Antalya, Turkey; 3 Akdeniz University Faculty of Medicine, Division of Gastroenterology, Antalya Turkey; 4 Akdeniz University Faculty of Medicine, Department of Cardiology, Antalya - Turkey

## Abstract

**Background:**

The role of inflammation in the pathogenesis and progression of atherosclerosis has been increasingly discussed. Although the seroepidemiological studies have suggested a relationship between Helicobacter *pylori *(*H. pylori*) infection and atherosclerosis; the issue is still controversial. It is well known that abnormal lipid profil is related to atherosclerosis and the measurement of carotid-intima media thickness (CIMT) is one of the surrogate marker of atherosclerosis. The serum concentration of high-density lipoprotein (HDL-C) has been known to have an inverse correlation with the development of atherosclerosis. Paraoxonase-1 (PON1) is a major anti-atherosclerotic component of HDL-C. PON1 activity is related to lipid peroxidation and prospective cardiovascular risk. The aim of this study was to investigate CIMT and serum PON1 activities along with lipid parameters in *H. pylori *positive and negative subjects.

**Methods:**

Thirty *H. pylori *positive subjects and thirty-one negative subjects were enrolled. *H. pylori *infection was diagnosed by the presence of positivity of stool *H. pylori *antigen test or Carbon 14 labeled urea breath test. Serum PON1 activity was measured spectrophotometrically. Traditional cardiovascular risk factors were investigated and laboratory analysis included measurement of serum triglycerides (TG), total cholesterol (TC), high-density lipoprotein (HDL-C) and low-density lipoprotein cholesterol (LDL-C). We assessed CIMT by high-resolution ultrasound of both common carotid arteries.

**Results:**

We found that the mean and maximum values of right and overall CIMT in *H. pylori *positive subjects were significantly thicker than those of *H. pylori *negative subjects. There was no significant differences in serum HDL-C, LDL-C, TC levels and TC/HDL-C ratios between two groups. Serum TG levels of *H. pylori *positive subjects were significantly higher than those of *H. pylori *negative subjects (p = 0.014). We found that PON1 activities were significantly lower in *H. pylori *positive subjects compared with negative subjects. No significantly correlation was observed between PON1 and CIMT values.

**Conclusions:**

There is an increase in CIMT values in patients with *H. pylori *positive compared to *H. pylori *negative subjects. PON1 activity decrease significantly in *H. pylori *positive subjects. However, an association between PON1 and CIMT was not found. These data indicated that *H. pylori *may have a role in atherosclerotic processes, however, further studies are needed to evaluate the exact mechanisms.

## Introduction

Helicobacter *pylori *is a gram negative curved bacillus that is frequently found in the human stomach and causes chronic and active gastritis, peptic ulcer disease and is associated with gastric adenocarcinoma [1,2]. Several studies have demonstrated that *H. pylori *infection is also associated with the development of coronary atherosclerosis [3]. Atherosclerosis pathogenesis includes abnormal lipid metabolism, endothelial dysfunction, inflammatory and immunological factors, plaque rupture and smoking [4]. Infectious processes can contribute to the pathogenesis of atherosclerosis by producing chronic infection of the vessels with inflammation leading to endothelial dysfunction [5]. It has been suggested that exposure to sustained high levels of endotoxin constitute a risk factor for atherosclerosis in animal models and, likely, in humans [5,6].

A low concentration of HDL-C is a well-known risk factor for coronary heart disease [5]. There are several well-documented functions of high plasma levels of HDL-C preventing the development of atherosclerosis. Most important function of HDL is to promote the efflux of cholesterol from cells. HDL-C also possesses antioxidant and antiinflammatory activities. The antioxidant activity of HDL-C is largely due to its paraoxonase-1 (PON1) content [7]. Human serum paraoxonase (PON1), a high-density lipoprotein (HDL)-bound ester hydrolase enzyme, has been shown to protect LDL from in vitro oxidation by hydrolysis of biologically active lipoperoxides [4]. Animal studies, including PON1-knockout mouse, demonstrated that PON1 deficiency was shown to increase susceptibility to LDL oxidation and atherosclerosis development [8]. In humans, it has been suggested that PON1 is significantly associated with atherosclerotic risk. PON1 activity is geneticly regulated and varies widely among populations [9]. Previous studies have shown LDL oxidation and the degradation of PON1 activity were significantly correlated [10]. Although serum PON1 activity in patients with coronary heart disease is decrease and is negatively related to the severity of coronary artery lesions, the correlation between PON1 activity and CIMT is controversial [11,12].

Existing cardiovascular diseases (CVDs) are found to be correlated with carotid artery intima-media thickness (CIMT) measured by ultrasound and are predictive of CVD in individuals without clinically evident disease. CIMT is now widely used as a early marker for atherosclerotic disease [13].

The aim of the present study was to evaluate the association between serum PON-1 activities and CIMT values in patients with *H. pylori*. For this purpose, we measured the CIMT and we compared the serum PON-1 activity and blood lipids between *H. pylori *positive and negative subjects.

## Materials and methods

### Patients

This study was approved by institutional ethical committee of Akdeniz University, Faculty of Medicine. The subjects were selected among adults who visited the Department of Gastroenterology because of dyspeptic symptoms and were tested for *H. pylori *infection in Akdeniz University Hospital, between August and November 2009. *H. pylori *infection was identified by the presence of positivity of *H. pylori *stool antigen test or Carbon 14 labeled urea breath test. We excluded patients who had acute infectious, rheumatologic and cardiovascular disease. For the study we included 30 *H. pylori *infected individuals (14 male and 16 female). 31 healthy noninfected, (15 male and 16 female), age and sex matched individuals were enrolled in this study for a control. All subjects were informed about the study and the written consent was obtained. We obtained detailed medical history about smoking habits, the presence of diabetes mellitus, hypertension, hyperlipidemia, family history of cardiovascular.disease and medication including antihypertensive and antihyperlipidemic drugs. Blood pressure was measured with manual sphygmomanometer. Body mass index (BMI; kg/m^2^) was calculated by dividing the body weight (kg) with squared height (m^2^). Their routine laboratory tests which contain complete blood count, serum glucose, creatinine and alanin aminotransferase (ALT) levels were recorded.

### Measurement of Serum Lipid and Lipoprotein Levels

Venous blood samples were obtained following an overnight fasting state. Serum samples were separated and stored at -80°C until the analysis. Serum total cholesterol (TC), HDL cholesterol (HDL-C) and triglyceride (TG) levels were measured with the enzymatic colorimetric method by using commercial kits on a Modular PPP auto-analyzer (Roche Diagnostics). Serum LDL-C levels were calculated by using Friedewald formula [14]. Serum TC/HDL-C ratio was also calculated. This ratio represents an atherogenic index, which is an important prognostic marker for cardiovascular disease [15].

### Measurement of Serum PON1 Activity

Serum PON1 activity was measured by adding serum to Tris buffer (100 mmol/L, pH 8.0) containing 2 mmol/L CaCl_2 _and 5.5 mmol/L paraoxon (O, O-diethyl-O-p-nitrophenylphosphate; Sigma Chemical Co). The rate of generation of p-nitrophenol was determined at 405 nm. according to MacKness B et al. [16]. The method was applied to an automated analyzer (Syva V-Twin, Siemens Diagnostics). The results are expressed as **U/L**.

### Ultrasound Scanning Procedure

Subjects were evaluated for carotid intima media thickness (CIMT) and plaque occurrence by using high resolution grey-scale Doppler ultrasonography. In a semi-dark room, all subject lay supine with their necks slightly hyperextended and rotated away from the imaging transducer. Both carotid arteries were scanned. CIMT was defined as the distance between the leading edge of the lumen intimal interface and the leading edge of the media adventitia interface of the far wall [13].

### Statistical Analysis

Data analysis was done with a statistical software package (SPSS for Windows, Version 16.0, SPSS Inc, and Chicago, Ill). Quantitative data were expressed as mean (± SD) or as medians. The comparisons of parameters were performed using Student's *t*-test. Correlation analyses were performed using Pearson's correlation test. A p-value of < 0.05 was considered as significant.

## Results

We included 30 subjects (14 male, 16 female) infected with *H. pylori *and 31 subjects (15 male, 16 female) without *H. pylori *infection. The demographic and clinical characteristics of study population are shown in Table 1. There were no statistically significant differences between two groups with regard to demographic and clinical characteristics (age, gender, BMI, smoking habits; history of diabetes mellitus; hypertension; family history for CVD).

**Table 1:** Demographic and Clinical Characteristics of Helicobacter *pylori *positive and negative subjects

Parameter	H. *pylori *positive subjects (n = 30)	H. *pylori *negative subjects (n = 31)	p value
Age (years)	40.9 ± 10.3	42.3 ± 9.4	NS

Gender M/F	13/17	14/16	NS

DM	1	2	NS

HT	4	4	NS

HPL	8	10	NS

Smoking	12	15	NS

Family history of CVD	3	4	NS

Systolic BP (mmHg)	119 ± 3	121 ± 8	NS

Diastolic BP (mmHg)	78.5 ± 8.8	79 ± 6.6	NS

BMI (kg/m2)	27.1 ± 3.7	26.2 ± 3.8	NS

Results are expressed as mean ± SD or number of patients.

**Abbreviations: **DM: diabetes mellitus, HT: hypertension, HPL: hyperlipidemia, CVD: cardiovascular disease, Systolic BP: systolic blood pressure, Diastolic BP: diastolic blood pressure. BMI: body mass index, NS: non-significant

As shown in Table 2; serum TG levels were significantly higher in *H. pylori *positive subjects than H. *pylori *negative subjects (1.81 ± 0.79 mmol/L vs 1.32 ± 0.73 mmol/L, p < 0.05). There were no statistically significant differences in TC, HDL-C, LDL-C levels and TC/HDL-C ratios between the two groups. Serum PON1 activity was significantly lower in *H. pylori *positive subjects when compared with negative subjects (270.03 ± 84.82 vs 340.00 ± 123.70 U/L, p < 0.05).

**Table 2:** Biochemical variables in Helicobacter *pylori *positive and negative subjects

Parameter	H. *pylori *positive subjects (n = 30)	H. *pylori *negative subjects (n = 31)	p value
Hb (g/dL)	13.6 ± 1.8	13.8 ± 1.5	NS

WBC (×10'/L)	7.38 ± 1.45	7.56 ± 1.98	NS

Glu (mmol/L)	5.44 ± 2.14	4.95 ± 0.67	NS

ALT (U/L)	28.4 ± 21	25.6 ± 23	NS

TC (mmol/L)	4.47 ± 1.32	4.69 ± 0.98	NS

LDL-C (mmol/L)	2.74 ± 0.80	2.84 ± 0.77	NS

HDL-C (mmol/L)	1.15 ± 0.30	1.21 ± 0.38	NS

TG (mmol/L)	1.81 ± 0.79	1.32 ± 0.73*	< 0.05

Cr (μmol/L)	63.64 ± 13.26	68.06 ± 9.72	NS

TC/HDL-C	4.13 ± 1.65	4.27 ± 1.95	NS

PON1 (U/L)	270.03 ± 84.82	340.00 ± 123.70*	< 0.05

**Abbreviations**: Hb: Hemoglobin, WBC: White blood cell, Glu: Glucose, ALT, Alanin amino transferase, TC: Total cholesterol, LDL-C: Low density lipoprotein- cholesterol, HDL-C: High density lipoprotein- cholesterol, TG: triglyceride, Cr: Creatinine, TC/HDL-C: Total cholesterol/HDL cholesterol, PON1: paraoxonase-1, NS: non-significant.

Results are expressed as mean ± SD. * p < 0.05 statistically significant

Structural measurements of vessels for *H. pylori *positive and negative subjects at enrollment are shown in Table 3. Atherosclerotic plaques in the common carotid artery were shown in 1% (3 of 30) of *H. pylori *positive patients and only 0.3% (1 of 30) of control subjects. The mean and maximum values of right CIMT were significantly increased in *H. pylori *positive subjects compared with negative subjects (p < 0.05). The mean and maximum values of left CIMT were tend to be higher in subjects with *H. pylori *but the differences were not statistically significant between two groups. We calculated mean and maximum overall CIMT by using left and right CIMT measurements. The mean and maximum values of overall CIMT were significantly higher in *H. pylori *positive subjects than negative subjects (p < 0.05) (Table 3).

**Table 3:** Structural and functional parameters of vessels for Helicobacter *pylori *positive and negative subjects

Parameter	H. *pylori *positive subjects (n = 30)	H. *pylori *negative subjects (n = 31)	p value
Mean Right CIMT (mm)	0.70 ± 0.09	0.64 ± 0.06	< 0.05

Max Right CIMT (mm)	0,81 ± 0,10	0.74 ± 0.07	< 0.05

Mean Left CIMT (mm)	0.72 ± 0.14	0.67 ± 0.08	NS

Max Left CIMT (mm)	0.83 ± 0.15	0.79 ± 0.1	NS

Mean Overall CIMT (mm)	0.71 ± 0.10	0.65 ± 0.06	< 0.05

Max Overall CIMT (mm)	0.82 ± 0.11	0.77 ± 0.01	< 0.05

Plaque	3	1	

**Abbreviations**: CIMT: Carotis intima-media thickness, NS: non-significant Results are expressed as mean ± SD or number of patients

* p < 0.05 statistically significant

In *H. pylori *positive subjects, serum PON1 activities were significantly correlated with HDL-C (r = 0.732, p < 0.05), with TG (r = -0.689, p < 0.05) and TC/HDL-C ratio (r = -0.334, p < 0.05). Serum HDL-C levels were significantly correlated with mean (r = -0.348, p < 0.05) and maximum (r = -0.366, p < 0.05) right CIMT, mean (r = -0.403, p < 0.05) and maximum (r = -0.403, p < 0.05) overall CIMT values while no correlation was found with mean and maximum left CIMT values (p > 0.05). We did not found any significant correlation between PON1 and CIMT values.

In *H. pylori *negative subjects, serum PON1 activities were significantly correlated with TG (r = -0.398, p < 0.05), HDL-C (r = 0.938, p < 0.05) and TC/HDL-C ratio (r = -0.628, p < 0.05). We did not find any significant correlation between serum PON1 activities and CIMT values (all p > 0.05).

## Discussion

A relation between atherosclerosis and chronic *H. pylori *infection was found in epidemiological studies [17-19]. Pellicano et al. reported significantly higher prevalence of *H. pylori *infection in patients with CAD than in controls (77% vs 59%) [20]. Although these studies have suggested a relationship between *H. pylori *infection and coronary heart disease; some of the underlying mechanisms still need to be discovered. It has been reported that chronic *H. pylori *infection results in decreased HDL-C levels, and these lipid alterations could, partially contribute to the initiation and development of coronary atherosclerosis [5,21,22]. Infection and inflammation are associated with a decrease in HDL-C levels. Induction of changes in lipoproteins by cytokines indirectly predisposes patients to atherosclerosis [23]. In the present study, we did not find any significant difference in TC, HDL-C, LDL-C levels and TC/HDL-C ratios between *H. pylori *positive and negative subjects. However, *H. pylori *positive subjects had significantly higher plasma triglyceride levels than negative subjects. Laurila et al. found significantly increased triglyceride (1.17 vs 1.00 mmol/L) and total cholesterol (6.34 vs 5.87 mmol/L) levels in 460 *H. pylori *positive subjects compared with 269 *H. pylori *negative subjects but HDL-C levels were found to be similar in both groups [24].

Major risk factors of atherosclerosis may explain only 50% of its etiology. Therefore, looking for new risk factors of atherosclerosis is necessary. HDL-C is a well known parameter inversely related to the risk for CVD. It plays a key role in the reverse cholesterol transport, protects LDL against oxidation and reduces lipoprotein associated peroxides. The antioxidant characteristics of HDL-C have been attributed to PON 1. In recent years, authors have suggested that low PON1 activity and concentration were important determinants of the presence of coronary artery disease [25]. Several factors like nutritional, pharmacological, genetic and infectious processes have been demonstrated to modulate PON1 levels [9]. Relationship between PON1 activity and infectious pathogens like HCV and HIV have been shown [26,27]. But, association between *H. pylori *infection and PON1 has been reported in only one study [4]. Aslan et al. found decreased serum PON1 activity in *H. pylori *positive patients [4]. In our study we also observed that serum PON1 activity was significantly lower in *H. pylori *positive subjects when compared with negative subjects.

There is considerable evidence suggesting that ultrasonic measurements of early atherosclerosis are clinically significant. In prospective studies increased IMT has been related to an increased risk of cardiovascular diseases [28,29]. There is conflicting data regarding CIMT and *H. pylori *infection. Some researchers have reported no relationship between *H. pylori *and CIMT [23,30,31]. However, Hamed et al. reported significant association between the two in diabetic patients [32]. In our study, the mean and maximum values of right and overall CIMT were significantly increased in *H. pylori *positive subjects compared with negative subjects. The mean and maximum values of left CIMT tended to be higher in *H. pylori *infected subjects but the differences were not significant between the two groups. We determined the correlations between PON1, HDL-C and CIMT respectively. While CIMT was negatively correlated to HDL-C, it showed no correlation with PON1. As is reported by Chen et al., HDL-C is a complex particle populated by multiple proteins that play a critical role in determining the overall effects of the lipoprotein [33]. Our results demonstrate that, PON 1 singularly could not represent the overall antioxidative activity of HDL-C.

The current study has certain limitations. Our study group was limited. We did not perform endoscopy and determine the grade of gastric inflammation or virulence factors of *H. pylori*.

In conclusion; serum PON1 activities were lower in *H. pylori *positive subjects while right and overall CIMT were significantly higher. Although we found no correlation between CIMT and PON1, these results implied that decreased PON1 activity may have an important role on vascular structural changes induced by *H. pylori *infection. Further studies with larger populations are needed to explore whether there is a strong relationship between PON1 activity and atherosclerosis in *H. pylori *infection.

## Competing interests

The authors declare that they have no competing interests.

## Authors' contributions

SHA participated in the design of study and performed the biochemical analysis. FD performed the statistical analysis. SB, IS, GS participated in the coordination of study. DK, SK, ID performed CIMT measurements. All authors read and approved the final manuscript.
